# Experiences and Perspectives of Polycystic Kidney Disease Patients following a Diet of Reduced Osmoles, Protein, and Acid Precursors Supplemented with Water: A Qualitative Study

**DOI:** 10.1371/journal.pone.0161043

**Published:** 2016-08-18

**Authors:** Jacob M. Taylor, Lauren Ptomey, Jill M. Hamilton-Reeves, Debra K. Sullivan, Catherine Creed, Susan E. Carlson, Donald E. Wesson, Jared J. Grantham, Cheryl A. Gibson

**Affiliations:** 1 Department of Dietetics & Nutrition, School of Health Professions, University of Kansas Medical Center, Kansas City, KS, United States of America; 2 Department of Nutrition Services, Children’s Mercy Hospital & Clinics, Kansas City, MO, United States of America; 3 Division of Cardiovascular Diseases, School of Medicine, University of Kansas Medical Center, Kansas City, KS, United States of America; 4 Department of Medicine-Nephrology, Kidney Institute, School of Medicine, University of Kansas Medical Center, Kansas City, KS, United States of America; 5 Department of Internal Medicine, Baylor Scott and White Health, Texas A&M Health Science Center College of Medicine, Temple, TX, United States of America; 6 Department of Internal Medicine, Division of General Medicine, School of Medicine, University of Kansas Medical Center, Kansas City, KS, United States of America; St Francis Hospital, UNITED STATES

## Abstract

**Background:**

Salt, protein, acid precursors, and fluid intake have been identified as factors that influence cyst growth in ADPKD. Unfortunately, the feasibility of following these dietary restrictions/enhancements from a patient’s point-of-view has yet to be studied. The purpose of this study is to understand better the experiences of patients following a relatively complex dietary prescription targeting these factors.

**Methods:**

Twelve adults with ADPKD and kidney function >30ml/min/1.73m^2^ were recruited from the University of Kansas Medical Center Polycystic Kidney Disease clinic. In a qualitative design, semi-structured interviews of participants were conducted following a four week dietary intervention (experimental diet lower in sodium, protein, and acid precursors, and supplemented with water) either face-to-face or by telephone. All interviews were recorded, transcribed verbatim, and checked for accuracy. Transcripts were analyzed thematically for emerging themes.

**Results:**

Participants reported that eating less meat and more fruits and vegetables were the easiest components of the diet, whereas reaching the daily goal amount of fruits and vegetables and tracking the diet constantly were the most difficult components. Participants had little difficulty with fluid intake and reported the prescribed fluid goal as achievable. The tracking system for fruits and vegetables and protein was reported to be both helpful and intuitive, but tracking their intake on paper was tedious. Eating out was the most significant barrier to following the diet with some individuals avoiding restaurants in order to comply with the dietary prescription.

**Conclusion:**

Participants on the experimental diet heightened their awareness of the consumption of dietary salt, protein, acid precursors, and fluid intake. Additionally, most participants believed adherence to the prescribed diet was feasible. However, participants wanted less cumbersome ways to track and monitor the diet, especially given that the prescribed diet is designed for lifelong adherence. Future studies should focus on targeting these specific dietary factors in larger groups of more ethnically and culturally diverse populations to help inform clinicians and how best to help diverse populations adhere to the dietary intervention.

**Trial Registration:**

ClinicalTrials.gov NCT01810614

## Introduction

Autosomal dominant polycystic kidney disease (ADPKD) is a genetic disorder characterized by the growth of cysts in the kidney and is the fourth leading cause of renal failure world-wide [1]. Dietary constituents including salt, protein, acid precursors, and fluid intake have all been identified as factors that influence cyst growth and progression to kidney failure in ADPKD [2–12]. Unfortunately, adherence to single component dietary prescriptions such as low sodium, low protein, or high fluid intake is relatively poor in the population as a whole, so adding more elements to the prescription would likely reduce long-term acceptance [13–16]. Moreover, given that damage to cystic kidneys for a lifetime, adherence to a dietary intervention must begin early and be a life-long commitment. Only recently has a composite dietary pattern reducing sodium, protein, acid precursors, and increasing fluid intake been prescribed, prepared, and followed by patients with ADPKD successfully; however, long-term adherence to this diet remains unstudied [17].

To improve treatment adherence and inform clinical practice, we performed a qualitative research study that expresses the views of individuals with ADPKD while following a diet designed to ameliorate disease progression. Understanding these views and experiences may help identify issues that would lead to long-term nonadherence, a behavior that would render the therapy ineffectual. To understand better the patient’s perspectives on following a relatively complex dietary prescription targeting dietary salt, protein, acid precursors, and fluid intake designed to slow disease progression, we interviewed individuals with ADPKD who had just completed a four week evaluation of a complex dietary prescription.

## Methods

### Design and Setting

A qualitative study was designed to follow a four week dietary intervention trial to lower the intake of sodium, protein, and acid-precursors and augment the intake of fruits, vegetables, and fluid [17]. Briefly, a comprehensive dietary history and two complementary 24h urine collections were analyzed to determine the intake and excretion of fluid, electrolytes, and protein on the subjects’ usual diet. At baseline subjects received instructions on how to modify their diet for the next four weeks. Each subject served as their own control in this pre-post feasibility study. The experimental diet was tailored for individual subjects based on their pre-study dietary intake of sodium, protein, fluid and net endogenous acid production (NEAP)(derived from three-day diet records) and measurements of urine osmolality and urine volume [18–20]. The test diet was followed for the next four-weeks which included limiting sodium to 1–1.5 mEq/kg, protein to 0.8–1.0 g/kg body weight/day (prescribed as points with 1 point equivalent to 7 grams of protein), consuming enough fruits and vegetables to reduce NEAP by 50% from baseline (prescribed as points with 1 point reducing NEAP 1 mEq), and drinking enough fluid to reduce mean urine osmolality to ≤ 285 mosm/kg H_2_O/day. The aims of the current study were to determine (1) what aspects of the diet were the easiest to follow, (2) what aspects of the diet were most difficult to follow, (3) barriers to following the diet, and (4) what can be done to improve the dietary regimen to promote long-term adherence. This study was approved by the Human Subject Committee at the University of Kansas Medical Center. Written informed consent was obtained prior to the start of the dietary intervention trial. This trial was registered at clinicaltrials.gov (NCT01810614).

### Data Collection

Face-to-face semi-structured interviews were conducted at the clinical and translational science unit, and, if not possible, phone interviews were conducted after the last study visit by an experienced, qualitative researcher with whom they had no previous contact. The semi-structured interviews allowed participants to describe their experiences of following the prescribed diet in depth and detail. This process was intended to enrich the findings from the trial that examined the effectiveness of the intervention. All interviews were conducted using an interview guide developed by the study team (S1 Table). Participants were asked semi-structured, open-ended questions about the diet and their overall perceptions about the dietary intervention. All interviews were recorded digitally and transcribed verbatim.

### Data Analysis

A thematic analysis was conducted from the transcripts of the semi-structured interviews to explore what components of the diet were the easiest to follow, what barriers made adherence to certain components difficult, and participants’ perspectives on areas for improvement that may improve long-term dietary adherence [21]. Interviews were reviewed by three researchers independently, and the interview guide was used as a coding framework for analysis [22]. Summaries were organized for each theme to facilitate discussion and analysis by team members [23]. Reliability and trustworthiness were established through discussion of any discrepancies among team members and re-examination of the transcripts was undertaken until consensus was achieved [24]. Specific quotes were chosen to represent themes that emerged from the transcripts. Participants did not contribute to data analysis or interpretation.

## Results

### Participant Characteristics

Twelve participants were enrolled in the dietary intervention trial and all participated in the interviews. Interviews ranged from 12 to 59 minutes (median, 28 min). The age range of participants was 22 to 65 years (mean, 48 years). The majority of participants were female (n = 7; 58%) and Caucasian (n = 10; 83%). Participants had a mean BMI of 25.03 kg/m^2^, mean blood pressure of 129/78, and had an estimated glomerular filtration rate of 83 +/- 22 ml/min/1.73m^2^ (mean +/- SD). Basic characteristics of each participant are included in Table 1.

**Table 1:** Demographic characteristics of 12 individuals with ADPKD who completed interviews after following a dietary intervention trial.

ID code	Age, y	Gender	Race	Marital Status	Family history of PKD	Diagnosis date	Stage of CKD
P1	57	Female	Caucasian	Married	No	1994	2
P2	62	Female	Caucasian	Married	Yes	1995	3A
P3	57	Male	Caucasian	Married	Yes	1993	3A
P4	61	Male	African American	Married	Unknown	2001	2
P5	22	Female	Caucasian	Single	No	1991	1
P6	50	Female	African American	Married	Yes	1996	2
P7	51	Female	Caucasian	Married	Yes	2002	1
P8	40	Male	Caucasian	Married	Yes	2011	1
P9	25	Male	Caucasian	Single	Yes	2003	1
P10	33	Female	Caucasian	Married	Yes	2010	1
P11	65	Male	Caucasian	Married	Yes	2010	3A
P12	58	Female	Caucasian	Married	Yes	2006	2

Abbreviations: y, year; ADPKD, autosomal dominant polycystic kidney disease; PKD, polycystic kidney disease; CKD, chronic kidney disease.

### Qualitative Findings

The six themes and quotes presented below represent the most salient features identified by the subjects.

#### Theme 1: Easiest components of the diet to follow

Participants (4 men, 4 women) reported that reducing their meat intake was much easier than they anticipated and for some was the easiest component of the diet to follow.

*“The easiest I guess probably cutting back on the protein was a pretty simple process*. *Just don’t eat meat*, *so that was*, *that was really easy*.*”*

(P7, Female, CKD Stage 1)

Participants also reported that eating more fruits and vegetables, especially those higher in points, was enjoyable and easy to follow (n = 6).

*“I actually really enjoy a lot of the higher point foods*. *So I think*, *shockingly*, *the vegetables were the easiest part for me once I realized what I needed in order to get my points for the day*.*”*

(P10, Female, CKD Stage 1)

One participant stated that reducing protein in the diet was easy, because the focus was on eating more fruits and vegetables, which essentially replaced meat at meals.

*“The focus was on [eating more] fruits and vegetables*, *so I did find it easy to reduce the protein*. *I’m not as big a meat eater as I thought*. *I didn’t miss it like I thought I would*.*”*

(P2, Female, CKD Stage 3A)

#### Theme 2: Most difficult components of the diet to follow

While participants reported eating more fruits and vegetables as one of the easiest components of the diet, half of the participants stated that reaching the amounts prescribed on the diet was one of the most difficult.

*“Just getting enough fruits and vegetables [was difficult] because I have such a high point amount that I usually just ended up picking the highest amount points of fruits and vegetables that I could find and I ended up eating a lot of like dried fruits and such*.*”*

(P5, Female, CKD Stage 1)

#### Theme 3: Ability to track the diet

With regards to the fruits and vegetables point system, several participants reported that it was intuitive and easy to understand (n = 8). A few participants did report some uncertainty when it came to tracking the number of points from particular fruits and vegetables since they were all standardized to half cup portions (n = 3).

*“I thought it was very*, *very easy*. *Especially since we have a scale at home*, *so it was pretty easy to measure out*. *The fruits and vegetable point system I thought was very*, *very easy and intuitive*.*”*

(P8, Male, CKD Stage 1)

*“Trying to figure out exactly how to measure things*. *The hardest thing for me to measure was a banana*.*”*

(P2, Female, CKD Stage 3A)

A few participants also stated that the protein point system was easy to follow with some reporting it as the easiest part of the diet (n = 3). Similarly, a few participants reported difficulty tracking the amount of protein using the point system, and preferred to measure protein in grams as an alternative, since it listed on most foods they ate (n = 3).

*“I think that part was probably the most simplest thing of all*.*”*

(P6, Female, CKD Stage 2)

*“It would have been easier to me to measure how many grams of protein that I’m supposed to eat instead of how many points*.*”*

(P2, Female, CKD Stage 3A)

Participants felt that reducing sodium was easy, but many did not track the exact amount consumed (n = 9). Participants reported avoiding high sodium foods by reading food labels, not adding salt to foods and using sodium free seasonings while cooking and at the dinner table, and eating at home more frequently to reduce sodium from processed foods and fast food restaurants.

*“Well I actually didn’t try to track the sodium much because the difference in the foods I was eating fresh fruits*, *fresh vegetables*, *with no sodium*. *There were very few things I was putting salt on or having sodium in it*.*”*

(P2, Female, CKD Stage 3A)

*“I did my best to reduce sodium*, *but it wasn’t something that I actively thought about too much*. *I think part of the reason that my sodium level went down was we were just eating more food at home and less take-out or whatever and that naturally just kind of reduces the sodium level*.*”*

(P8, Male, CKD Stage 1)

Most participants reported that while they had to drink more fluid on the diet, it was an attainable amount (n = 9). Participants primarily reported two different ways of tracking fluids to account for the amount of fluids consumed. One way was using a water bottle of a specific size and another means was to track the number of bottles consumed each day. Participants stated that when they struggled to reach their water intake goal it was because they had forgotten to take their water bottle with them.

*“I was already drinking quite a bit*. *I’ve made that a goal more recently to drink more because I know it’s better for me*, *so I didn’t really add much more for this diet to what I was drinking before*. *But yeah*, *the only time I really do struggle is if I don’t have my water bottle on me or access to any fluids*.*”*

(P5, Female, CKD Stage 1)

*“I had a couple of water bottles that I kept in the fridge that were*, *you know*, *a specific amount*. *And then I just tracked the water bottles were 20 ounce each*, *so I just kept trying to make sure that I always had one going and*, *you know*, *where I could see it and then I would just make the effort to drink from it*.*”*

(P7, Female, CKD Stage 1)

Participants reported that certain aspects of tracking were difficult to do. Difficulties tracking were primarily related to keeping a daily tally of all foods and beverages they were consuming. While participants were able to track their dietary intake over the course of the study (4 weeks), most participants reported that they would not be able to track it using paper documents over the course of their lifetime. Keeping a daily log and tallying their dietary intake manually was described as too much of a burden to continue lifelong (n = 10). However, participants reported that they would strive to continue following the dietary themes consistent with the goals set by the prescribed diet (eating less sodium and protein, and consuming more fruits, vegetables, and fluid) after completion of the study.

*“Most difficult [was] probably having to track every day*, *every single day*. *So for me*, *[it was] documenting it all*.*”*

(P1, Female, CKD Stage 2)

*“I’m not so much going to track it by points*, *but just make a conscious effort to eat less protein and more fruits and vegetables just all the time*.*”*

(P9, Male, CKD Stage 1)

*“Well*, *I’m trying to kind of follow it somewhat*. *Well*, *just eat more fruits and vegetables and not eating so much meat and drinking lots of water because I know I need lots of fluids*. *No*, *I’m not really tracking it*.*”*

(P3, Male, CKD Stage 3A)

#### Theme 4: How diet affected other aspects of life

Participants reported a change in the way that they went grocery shopping with an emphasis on reading food labels more closely and selecting more fruits and vegetables, particularly those that were higher in points.

*“I definitely spent a lot more time in like the fresh produce area of the grocery store than the rest of it*. *That was mostly what my diet was made up of*.*”*

(P5, Female, CKD Stage 1)

*“If you take the list with you of the points it definitely made it a lot easier*. *I was like*, *oh hey*, *this is worth a lot of points*, *I’ll pick this up*. *It just made it a lot easier to carry that with me*.*”*

(P9, Male, CKD Stage 1)

Some participants avoided eating out due to the uncertainty of what they could eat (n = 2), whereas others reported spending more time looking at online nutrition facts prior to going out, so that they could make the most appropriate selections (n = 3).

*“We didn’t*. *I mean we did a couple times when I had the events planned but there were several times when we said oh*, *I guess we can’t do that*. *[When we did go out]*, *I would look at Sheridan’s versus McDonalds ice cream/custard to see which one was better and we’d go to that one*.*”*

(P1, Female, CKD Stage 2)

In order to continue to adhere to the diet and ensure that appropriate foods would be available, two participants reported taking food with them to social events (n = 2).

*“You know*, *sometimes at a restaurant or at somebody else’s home when their serving food or whatever*, *they may not have what I should be eating*. *A lot of times I brought my own stuff*.*”*

(P7, Female, CKD Stage 1)

#### Theme 5: Barriers to following the diet

Attending social events, traveling, and dealing with family medical issues made it particularly difficult for participants to adhere to the prescribed diet. Most participants had to attend at least one social event during the four weeks on the diet or had to travel for personal or business reasons (n = 7). Participants reported this was the most likely time for them to not follow the diet due to limited availability of food or having to eat out more frequently where no ideal options were available.

*“I went out of town one day and I couldn’t really concentrate on the diet that day because I was out of town and I was busy*. *That day was hard*. *Going out of town made it really*, *really hard*.*”*

(P12, Female, CKD Stage 2)

Two individuals reported having to deal with family medical issues where taking care of others took precedent over following the diet at those times.

#### Theme 6: Suggestions for improving diet

Overall, eleven of the twelve participants were able to adhere to the diet over the course of the dietary intervention with eight participants reporting being able to adhere to the diet over a much longer period of time. However, participants suggested the need for a phone app to mitigate the challenges of tracking and tallying what was consumed over the course of the day (n = 3).

*“[Use of] a possible phone app*. *I mean that would probably work*. *I think I could do it that way just cause I’m always around my phone*.*”*

(P5, Female, CKD Stage 1)

*“Most people have phones that you can take notes on or I would think*. *There might already be a food tracking app where you just like plug in as you go throughout your day*. *I think that would’ve been helpful*.*”*

(P10, Female, CKD Stage 1)

Participants also reported that additional recipes or meal/snack ideas that fit the diet would have been beneficial, making the initial transition to the prescribed diet easier. Additionally, one participant suggested that a gradual progression into each targeted component of the diet might have helped make the initial transition less difficult.

*“Like I don’t really know what’s out there that I could make*, *so maybe more like recipes ideas or something like that or what I can make and*, *you know*, *how to best get the higher points and stuff*. *I think that would help*.*”*

(P5, Female, CKD Stage 1)

*“I think it would be better*, *if I were having to do this*, *to start with one item*. *Like let’s start this week with protein*. *We want you to cut back your protein to this*. *Now you can eat as many fruits and vegetables as you want*, *but cut back your protein and learn to eat less protein*. *So get that down and then start looking at your sodium*. *And then start*, *you know what I mean*? *I think I would do it gradually because to just do it all at once was a complete diet change for me and I am a pretty healthy eater*. *I mean I really am*, *so for me it was still a transition so I can’t imagine what it’s like for someone who is a real meat and potatoes*, *eat[er] outer*, *it would be hard*.*”*

(P1, Female, CKD Stage 2)

## Discussion

PKD is the most common genetic cause of kidney failure, yet, these individuals remain an understudied population in need of additional research to determine interventions that may reduce the severity of ADPKD and preserve native kidney function. This study looked at a small group of individuals with ADPKD who had just completed a four week evaluation of a complex dietary prescription to determine their views, experiences, and whether this type of therapy could be followed. There was general consensus that reducing portion sizes of meat and increasing intake of fruits and vegetables were the easiest components of the diet while keeping track of what they ate and reaching the prescribed goal amount for fruits and vegetables each day were the most difficult components. Participants thought the tracking system (points) for fruits and vegetables and protein was intuitive and easy to use. Although a few participants commented that some fruits and vegetables were difficult to measure since they were all measured in ½ cup portions (ie. Banana), and some participants would have liked the option to measure protein in grams. Participants didn’t track sodium intake, but instead avoided high sodium foods, salt-based seasonings, and limited eating at restaurants. Water intake was tracked by counting water bottles of a specific size to account for fluid consumed throughout the day and was reported as an attainable amount. Lastly, participants reported a change in how they shopped at the grocery store and that the biggest barrier to following the diet involved being away from home (attending social events and traveling).

Reduction of salt is essential for patients with ADPKD due to the effects sodium has on raising blood pressure and stimulating the production of vasopressin, factors that likely accelerate the development of cysts in the kidneys [3, 25–27]. Previous studies in patients with chronic kidney disease have shown that avoiding use of salt in cooking or at the table and eating fast food less frequently are more frequent behaviors in patients who report self-adherence to a low sodium diet [28]. In our study, participants were able to reduce sodium citing these same behaviors are the primary ways they achieved reducing sodium in their diet. While, in general, adherence to a low sodium diet is poor, the employment of general sodium reduction guidelines (reducing salt and sodium-based seasonings and limiting eating at restaurants) seems to be effective at lower sodium consumption in individuals with ADPKD [16, 17].

Consumption of animal-sourced protein also plays a role in the growth of cysts in patients with ADPKD, making protein restriction a viable therapy for ADPKD [3, 29]. In the MDRD study, people who were able to adhere to a low-protein diet were more likely to self-monitor intake more frequently and had better adherence when they had non-protein options to replace energy intake [30]. Our dietary intervention used similar strategies which included self-monitoring protein intake daily and replacing some of their meat intake with fruits and vegetables. Participants not only were able to reduce their consumption of protein, but even reported it as one of the easiest components of the diet to follow. Focusing on increasing consumption of fruits and vegetables as a way to take the focus away from meat as the primary entrée seems an effective and easy strategy to reduce protein intake even with typical Midwestern meat-laden diets.

Higher levels of acid excretion have shown to accelerate disease progression in an animal model of ADPKD, while the administration of alkali was able to reverse the damage [2, 5]. Recent studies of chronic progressive renal insufficiency in patients with hypertensive nephropathy approaching the end-stage of the disease have exposed the harmful effects of hydrogen ion excretion in patients eating regular diets containing abundant acid precursors [6, 31]. Potassium aids in the renal excretion of dietary acids, and may be kidney protective, giving fruits and vegetables an important place in a variety of kidney diseases, including ADPKD. Patients in our study were able to increase consumption of fruits and vegetables for four weeks and stated that it was one of the easiest components to follow. However, participants did report issues with consuming the prescribed number of points from fruits and vegetables. Some participants made fruit smoothies in the mornings to secure a large portion of their points for the day to avoid consuming large quantities of fruits and vegetables in the evening to reach their prescribed points. The extent to which dietary acids need to be reduced is not known; however, we think that lowering urinary acid excretion in ADPKD to any extent below the subject’s usual will be as helpful over the long run.

Vasopressin stimulates cyst growth in ADPKD and an inhibitor targeting the vasopressin receptor (AVP-2) has been shown to slow disease progression [32–34]. Increasing the intake of water might also have a similar effect on cyst growth [12]. Participants reported that the amount of fluid prescribed on this diet was attainable and that the only barrier to achieving their intake was making sure they had their water bottle with them throughout the day. Patients with ADPKD are often instructed to treat fluid like a prescribed medication given its potential to slow cyst growth and in our dietary intervention the dose likely to lower vasopressin secretion to minimal amounts proved to be achievable by patients. Physicians should be discussing fluid intake with both adults and children diagnosed or presumed to have ADPKD, since patients even early in the course of the disease appear to be adherent to drinking fluids above the usual amount they would consume.

While participants were able to adhere to the diet for four weeks, most participants reported that while they would continue to try to follow the diet, they would not continue to track or maintain the prescribed dietary goals. Although participants indicated a greater awareness of their food and beverage consumption, they remarked that substantial changes required by the prescribed diet were not sustainable. Participants primarily remarked that tracking and monitoring the diet was tedious and something they would stop doing, especially since it needs to be done on a daily basis. The use of apps for dietary changes has shown promise in getting patients to adhere to dietary changes, but most notably it has the ability to improve the willingness to stay on the diet long-term [35]. Given that PKD is a slow progressing chronic disease, dietary changes would need to be prescribed at a very young age and be followed over the course of their lifetime. The development of an app specifically designed to track sodium, protein, dietary acid load, and fluids could facilitate improving lifelong adherence to this diet; however, use of an app may not be appropriate or useful for all patient populations. Further studies are needed in different age groups, ethnicities, and races to determine what other methods may be suitable to improve adherence to the dietary treatment in those populations. Notwithstanding, given the regular follow-up for patients with progressive renal disorders, we think that positive encouragement to follow the PKD diet by renal dietitians during clinic visits can help educate patients on the importance of following this diet, potentially help improve adherence, and slow the progression of the disease.

Limitations in this study include the small sample size and enrollment of primarily middle-aged, Caucasian men and women with well-preserved kidney function. Due to the limited demographic profile of the participants enrolled into our dietary intervention trial, the qualitative results may not be representative of those individuals under 18 years of age, individuals further along in the course of the disease, or individuals from different cultures and ethnic backgrounds. As diet is largely influenced by culture, our findings may not be generalizable to the same burdens individuals may face while trying to follow the prescribed dietary pattern in our study. Additionally, interventions aimed at improving adherence to the diet among racially and ethnically diverse groups may be different than what we have derived from our limited sample of individuals living in the Midwest region of the United States. Despite these limitations, a qualitative study examining the perceptions following a relatively complex prescription was needed to determine if individuals with ADPKD thought they could follow this diet long-term and if modifications to the diet were needed. The primary strength of this study is that it is the first qualitative study conducted in individuals with ADPKD following a diet specifically designed to ameliorate ADPKD progression. Additionally, the implementation of our newly developed point system for fruits and vegetables for tracking NEAP was found to be intuitive and easy to follow by most study participants. Indication for continued use in dietary trials aimed at reducing dietary acids seems reasonable.

## Conclusion

This is the first qualitative study conducted in individuals with ADPKD following a composite dietary intervention that aims to address the dietary components that may accelerate disease progression. Exploring patient views on the prescribed diet provided valuable information for clinical practice and insight on how patients’ managed their diets to slow the progression of ADPKD. Overall, participants in this study felt positive about making dietary changes, willing to continue to decrease the consumption of sodium and protein, increase the consumption of fruits, vegetables, and fluids, and became much more aware of their dietary behaviors. Participants reported being able to reduce sodium, restrict protein intake, increase fruit and vegetable intake, and consume the appropriate amount of fluids with ease. The primary barriers to following the diet were attending social events and traveling. Participants reported that tracking their diet was tedious and that improvements should be focused on improving that aspect of the dietary regimen. This study demonstrates that while patients are able to adhere to a low-sodium, low-protein diet attenuated with fruits, vegetables, and fluids in the short-term, improving how they track and monitor their intake is necessary if planning to adhere to the diet over one’s lifetime.

## Supporting Information

S1 File Consolidated criteria for reporting qualitative studies (COREQ): 32-item checklist. (DOCX)

S2 File TREND Statement Checklist. (PDF)

S3 File ADPKD Pilot Study Protocol. (DOCX)

S1 Table Semi-structured interview questions. Abbreviations: PKD, polycystic kidney disease.
(DOCX)
