# Supplementary material for: Experiences and Perspectives of Polycystic Kidney Disease Patients following a Diet of Reduced Osmoles, Protein, and Acid Precursors Supplemented with Water: A Qualitative Study
Source: PLoS One. 2016 Aug 18;11(8):e0161043. doi: 10.1371/journal.pone.0161043 (PMC4990231; doi:10.1371/journal.pone.0161043)
Supplement: S1 File — (DOCX) [file pone.0161043.s001.docx]

Consolidated criteria for reporting qualitative studies (COREQ): 32-item checklist

| **No** | **Item** | **Guide questions/description** |
| --- | --- | --- |
| **Domain 1: Research team and reflexivity** |  |  |
| Personal Characteristics |  |  |
| 1. | Interviewer/facilitator | Which author/s conducted the interview or focus group?  LP conducted the interviews |
| 2. | Credentials | What were the researcher's credentials?  MS, RD, LD |
| 3. | Occupation | What was their occupation at the time of the study?  Dietitian and PhD candidate |
| 4. | Gender | Was the researcher male or female?  Interviewer was female. |
| 5. | Experience and training | What experience or training did the researcher have?  Interviewer had previous experience in conducting semi-structured interviews and Senior investigator is an associate professor with experience conducting focus groups and qualitative research in weight management behaviors. |
| Relationship with participants |  |  |
| 6. | Relationship established | Was a relationship established prior to study commencement?  Interviewer had not met patients prior to interview. |
| 7. | Participant knowledge of the interviewer | What did the participants know about the researcher?  Participants knew interviewer worked on study with PI, and was aware of the diet they had followed, but not involved in the creation or influenced by outcome of the diet. Participants were allowed to express themselves freely as they had no previous engagement with interviewer and would have been less inclined to tell her what they thought she wanted to hear. |
| 8. | Interviewer characteristics | What characteristics were reported about the interviewer/facilitator?  That she had not met participants prior to conducting interviews. This was done in an effort to reduce bias. |
| **Domain 2: study design** |  |  |
| Theoretical framework |  |  |
| 9. | Methodological orientation and Theory | What methodological orientation was stated to underpin the study?  This is a qualitative study using semi-structured interviews to allow for participants to share experiences in various areas after following a low sodium, low protein, low acid precursor diet, enhanced with fluid. |
| Participant selection |  |  |
| 10. | Sampling | How were participants selected?  Patients with ADPKD were approached in a PKD clinic about participating in a dietary intervention trial for PKD. |
| 11. | Method of approach | How were participants approached?  Participants were approached face-to-face in clinic |
| 12. | Sample size | How many participants were in the study?  ~50 were approached about enrolling and 12 enrolled. |
| 13. | Non-participation | How many people refused to participate or dropped out? Reasons?  ~38 did not want to participate and after we had 12 enrolled, all participants finished study. |
| Setting |  |  |
| 14. | Setting of data collection | Where was the data collected?  Interviews were conducted in research study room or via phone if patient unable to do interview after last study visit. |
| 15. | Presence of non-participants | Was anyone else present besides the participants and researchers?  No. Just researcher and participant. |
| 16. | Description of sample | What are the important characteristics of the sample? Important characteristics presented in Table and include age, gender, race, marital status, family hx of PKD, diagnosis date, and CKD stage |
| Data collection |  |  |
| 17. | Interview guide | Were questions, prompts, guides provided by the authors? Was it pilot tested?  Yes (supplemental table). Semi-structured interview guide adapted from previous studies interviewer had been involved in. PI, SI, and interviewer agreed on questions |
| 18. | Repeat interviews | Were repeat interviews carried out? If yes, how  many?  No. |
| 19. | Audio/visual recording | Did the research use audio or visual recording to collect the data?  Yes, all audio recorded for later transcription. |
| 20. | Field notes | Were field notes made during and/or after the interview or focus group?  Interviewer took field notes during interview. |
| 21. | Duration | What was the duration of the interviews or focus group?  12 to 59 minutes (median, 28 min) |
| 22. | Data saturation | Was data saturation discussed?  No. Dietary intervention was pilot study so all participants were asked to complete interview. |
| 23. | Transcripts returned | Were transcripts returned to participants for comment and/or correction?  No. |
| **Domain 3: analysis and findings**z |  |  |
| Data analysis |  |  |
| 24. | Number of data coders | How many data coders coded the data?  3 |
| 25. | Description of the coding tree | Did authors provide a description of the coding tree?  None provided in study. |
| 26. | Derivation of themes | Were themes identified in advance or derived from the data?  Themes were identified from data. |
| 27. | Software | What software, if applicable, was used to manage the data?  Data audio recorded on freeconferencecall.com then uploaded then stored on KUMC servers. |
| 28. | Participant checking | Did participants provide feedback on the findings?  No. Findings were not available at the time of interviews and not sent to participants until after publication. |
| Reporting |  |  |
| 29. | Quotations presented | Were participant quotations presented to illustrate the themes / findings? Was each quotation identified?  Yes. |
| 30. | Data and findings consistent | Was there consistency between the data presented and the findings?  Yes. |
| 31. | Clarity of major themes | Were major themes clearly presented in the findings?  Yes. |
| 32. | Clarity of minor themes | Is there a description of diverse cases or discussion of minor themes?  Given relatively small sample size, only major themes discussed. |
