# Supplementary material for: Experiences and Perspectives of Polycystic Kidney Disease Patients following a Diet of Reduced Osmoles, Protein, and Acid Precursors Supplemented with Water: A Qualitative Study
Source: PLoS One. 2016 Aug 18;11(8):e0161043. doi: 10.1371/journal.pone.0161043 (PMC4990231; doi:10.1371/journal.pone.0161043)
Supplement: S3 File — (DOCX) [file pone.0161043.s003.docx]

**ADPKD Pilot Study Protocol**

**Study Title: A new diet for patients with autosomal dominant polycystic kidney disease (ADPKD).**

**Principal Investigator: Jared J. Grantham M.D**

Department of Medicine/Kidney Institute

jgrantha@kumc.edu

**Co-investigator: Jacob Taylor, MS, RD, LD**

Department of Dietetics and Nutrition

jtaylor10@kumc.edu

**Co-investigator: Jill Hamilton-Reeves, PhD, RD, LD**

Department of Dietetics and Nutrition

jhamilton-reeves@kumc.edu

**Co-investigator: Debra Sullivan, PhD, RD**

Department of Dietetics and Nutrition

dsulliva@kumc.edu

**Co-investigator:** **Cheryl Gibson, PhD**

Department of Internal Medicine

cgibson@kumc.edu

**Sponsor: University of Kansas Medical Center**

**Division of Nephrology**

**PROTOCOL:**

**APPROVALS:**

**A. GOALS AND SPECIFIC AIMS:**

Six hundred thousand people in the United States and 12.5 million people worldwide are affected by Polycystic Kidney Disease (PKD) (1). A specific form of PKD termed Autosomal Dominant Polycystic Kidney Disease (ADPKD) occurs in between 1 in 400 to 1 in 1000 people (2). ADPKD is a disease defined by **cysts that develop in individual renal tubules and enlarge progressively throughout life causing kidney volume to increase. Recent evidence has shown that kidney volume predicts the likelihood of developing renal insufficiency over a finite length of time, suggesting a linkage between the growth of cysts and the harm they do to kidney function (3). Moreover, the rate of renal enlargement appears to be tied to dietary factors, since total kidney volume associated positively with body surface area, and total urinary sodium, urea, and total solute excretion (4,5).**

**S**tudies conducted by Wesson, et al. have demonstrated that reduced urinary acid excretion through the addition of dietary fruits and vegetables or orally administered sodium bicarbonate/sodium citrate can attenuate kidney injury markers and better preserve GFR in patients with hypertensive nephropathy (6,7,8). This raises the possibility that dietary acid precursors might have a similar adverse effect in polycystic kidneys.

The short-term goal of this pilot study is to determine the extent to which the Kansas PKD diet reduces net acid excretion in individuals with ADPKD and to judge if that diet is palatable and sustainable.

The long-term goal is for the Kansas PKD diet to be adopted by physicians and dietitians for administration to patients early in the course of the disease. Positive outcomes in this pilot study are key to moving forward to long-term, large scale studies to test whether the diet ameliorates disease progression in individuals with ADPKD who have good to excellent renal function.

The over-riding hypothesis guiding this study supposes that dietary constituents contributing to net acid excretion have an adverse effect to accelerate the development and growth of renal cysts and, thereby, promote the deleterious impact of the cysts on structures that determine life-sustaining renal function. Reduction in the renal excretion of acid will ameliorate disease progression evinced by the stabilization of glomerular filtration rate (GFR) and prolongation of life-sustaining renal function.

**Specific Aims:**

The specific aims of this pilot study are:

- **Aim 1- To determine net acid excretion in individuals consuming the Kansas PKD diet in comparison to their usual diet.**
  - We *hypothesize* that the Kansas PKD diet will decrease the rate of net acid excretion.
- **Aim 2- To evaluate the acceptance and sustainability of the Kansas PKD diet**
  - We *hypothesize* that a diet composed of diminished net acid precursors will be accepted by participants and sustainable.
- **Aim 3-To determine if the Kansas PKD diet reduces kidney injury markers in comparison to the individual’s usual diet.**
  - We *hypothesize* that the Kansas PKD diet will reduce urine markers of renal injury in participants.

**B. Background and Significance:**

**Recent evidence has shown that kidney volume predicts the likelihood of developing renal insufficiency over a finite length of time in ADPKD, suggesting a linkage between the growth of cysts and the harm they do to kidney function (3).** This evidence indicates that cysts may cause injury to key structures involved in generating the formation of glomerular filtrate, leading to early compensation for decreasing nephrons by elevating filtration rates in the surviving glomeruli (9). Consequently, injuries are occurring decades before an absolute decline in GFR can be measured and are currently going untreated. Eventually, t**his decline in kidney function will result in** end stage renal disease (ESRD) with recent data suggesting that between 50 percent and 60 percent of patients with ADPKD will have kidney failure by age 60 and age 70, respectively (2). Improving clinical care that can prolong kidney function needs to be examined to improve outcomes in ADPKD patients.

**Recent studies indicate that the rate of kidney volume increase is hastened by excess dietary protein, salt, and potential net acid precursors, and slowed by increased water intake sufficient to lower plasma vasopressin levels (4,5). Diets are commonly prescribed to treat ADPKD and other renal patients with disease near the end-stage, but there is currently no specific diet prescription that takes potentially harmful dietary elements into account for ADPKD patients in the earliest stages of the disease. S**tudies conducted by Wesson, et al. have demonstrated that reduced urinary acid excretion through the addition of dietary fruits and vegetables or orally administered sodium bicarbonate/sodium citrate can attenuate kidney injury markers and better preserve GFR in patients with hypertensive nephropathy (6,7,8). These studies indicate that H+ ions play a role in damaging renal tubules accelerating the rate that kidney function declines, although the exact mechanisms are still unknown. Diet plays a major role in the establishment of acid/base balance and certain dietary components actuate the production of H+ ions (10). Lacking in the PKD field are studies to examine how dietary interventions can slow the progression of the disease by reducing kidney injury. In this regard, I have devised a novel diet for ADPKD, from this point to be referred to as the Kansas PKD diet, which I believe will reduce net acid excretion and kidney injury markers, thereby slowing disease progression in ADPKD patients. **This diet is the first to be directed at preventing the growth of cysts in ADPKD patients.**

I propose a pilot study to verify that the Kansas PKD diet prescription will reduce net acid excretion (NAE) below the levels that participants excrete while eating their usual diets. NAE is equivalent to the difference between the urinary excretion of anions and cations and is equivalent to the sum of ammonium (NH_4_) and titratable acid (TA) - bicarbonate measured in urine. When the usual urinary electrolyte anions exceed electrolyte cations, NAE is positive and the urine pH is acidic, and when usual electrolyte cations exceed anions, NAE is negative and urine pH is alkaline (6-8,10). We expect that normal individuals in Kansas City (a city with heavy emphasis on meat-laden diets) will have potential renal acid load (PRAL) of ~ 60 – 80 mEq/day and, therefore, a positive NAE of ~ 60 – 80 mEq/day (11).

Significance: This pilot study will be an important step toward validating a diet that reduces net acid excretion (NAE) in participants with ADPKD. If it is successful, there is reason to expect that a full-scale, controlled clinical trial would be undertaken to determine the extent to which dietary limits on sodium, net acid excretion and total solutes, together with a modest increase in the daily intake of water, would improve outcomes in ADPKD patients.

**C. Preliminary Studies:**

**As mentioned above, recent studies in humans and experimental animals with cystic kidneys indicate that the rate of kidney volume increase is hastened by excess dietary protein, salt and total urinary solutes (osmolar excretion) and slowed by increased water intake sufficient to lower plasma vasopressin levels (4,5). S**tudies conducted by Wesson, et al. have demonstrated that reduced urinary acid excretion through the addition of dietary fruits and vegetables can attenuate kidney injury markers and better preserve GFR in patients with hypertensive nephropathy (6,7,8). These studies indicate that excreted H+ ions are capable of injuring renal tubules and contribute to the rate that kidney function declines. The specific mechanisms by which excess protons injure renal tissue is unknown, but under active study. Interestingly, Cowley et al showed many years ago that the administration of ammonium chloride, increased dietary protein (casein), or severe potassium depletion to rats with a type of ADPKD increased the rate of disease progression (12). The urinary excretion of acid is known to increase after the administration of ammonium chloride, or the feeding of a high protein or low potassium diet suggesting that protons may have had an adverse role in this disease model. Tanner and colleagues have validated the effects of ammonium chloride in the rodent model of ADPKD and have shown that the administration of alkalinizing salts ameliorates disease progression (13).

It is well known that diet plays a major role in the determination of acid/base balance (10), however, no studies have been done in individuals with ADPKD to determine the extent to which dietary constituents effect disease outcomes. In this regard, the Kansas PKD diet was devised specifically to target dietary constituents associated with increased kidney volume and adding to potential renal acid load (PRAL) in ADPKD patients. I expect that individuals in Kansas City (heavy emphasis on meat-laden diets) will have potential renal acid load (PRAL) of ~ 60 – 80 mEq/day and, therefore, a positive NAE of ~ 60 – 80 mEq/day (11). I believe the Kansas PKD diet will reduce net acid excretion by 50% from participants usual dietsand, thereby, potentially decrease kidney injury.

**D. Research Design and Methods**

Study population: This pilot study will include adult subjects (age ≥ 18years) with a certain diagnosis of ADPKD confirmed by magnetic resonance, computed tomography or ultrasound imaging (14) in a sequential design in which each subject is used as his/her own control. I will enroll a total of 10 subjects (5 women, 5 men) who have KDOQI Stage 3 renal function or better (eGFR > 30ml/min/1.73m2) based on the MDRD-EPI equation. We may need to recruit more than 10 subjects in the event that the NAE requirement is not met or we have drop outs from the initial recruitment. All patients who qualify for the study, regardless of gender, race/ethnicity, etc, will be offered the opportunity to participate. Participants will be recruited from KUMC Nephrology clinic which has an adequate patient population that meets inclusion/exclusion criteria. Initial contact will be made by the patient’s treating Nephrologist duringroutine clinic visits. Interested patients will be contacted by a member of the KUMC study team. Dr. Grantham, staff nephrologists or study coordinators will consent the patients during study visits at the CTSU or KUMC clinic. Consent interviews will take place in a private clinic room. Subjects will have the opportunity to read the consent forms in the absence of the PI and study team. It will be explained to the subjects that the study is voluntary and that they may discontinue at any time without prejudice. Subjects will have the opportunity to ask questions before signing the consent form.

The HERON data base will also be used to find eligible patients who have previously given permission to be contacted about research studies. The coordinators will contact PKD patients who qualify and determine if they are interested. Subjects will be receive a total of $200 to compensate for time and travel.

**Inclusion criteria**

a. Ten healthy individuals with ADPKD who have not been prescribed dietary restrictions or enhancements.

b. Blood pressure <135/85 mmHg with or without specific treatment.

c. Willingness to participate for at least 5 weeks.

d. Diet history consistent with the excretion of >30 mEq NAE / day- based on usual dietary intake (collected at baseline visit using diet records and NDSR software calculations and to be confirmed with urine sample analysis of urinary acid excretion).

**Exclusion criteria**

a. Other active diseases requiring pharmacologic agents

b. Unstable weight (±2 kg) for 3 months prior to enrollment

d. Pregnancy or lactating

e. Confounding medications, i.e. bicarbonate, citrate

f. Individuals who do not consume meat for personal, religious, or health reasons.

**Design**

**Table 1. Summary of experimental protocol and general study design**

| **Enrollment Visit** | **Days 1-7** | **Baseline Visit (day 8)** | **Days 8-21** | **Interim visit (day 22)** | **Days 22-35** | **Final Visit (day 36)** |
| --- | --- | --- | --- | --- | --- | --- |
| Informed consent | Eat  Baseline  Diet | Collect urine, serum | Eat  Experimental  Diet  Phone call follow-up on Day 14 | Collect urine, serum | Eat Experimental Diet  Phone call follow-up on Day 28 | Collect urine, serum |
| Medical history |  | Analyze diet record |  | Analyze diet record |  | Analyze diet record |
| Distribute materials |  | Compute PRAL |  | Compute PRAL |  | Compute PRAL |
| Begin baseline diet |  | Instruct in Kansas PKD diet |  | Reinstruct in Kansas PKD diet if needed |  | Conduct Semi-structured interviews and Nutrition Hassles Questionnaire |
| Collect height, weight, and BMI measurements |  | Collect height, weight, and BMI measurements |  | Collect height, weight, and BMI measurements |  | Collect height, weight, and BMI measurements |

**Enrollment visit.**

a. Explain project in detail and obtain signed and witnessed informed consent.

b. Record medical history emphasizing nutrition; Perform limited physical exam including measurement of blood pressure, height and weight.

c. The enrollment period will begin on a Thursday or Friday and will last one week during which the subjects eat their usual diets and fill out diet records on days 5, 6 and 7. This is to avoid collecting urine samples during the weekend when the diet may be more variable

d. Instructions on how to collect complete 24h urine samples will be given; samples will be collected on days 6 and 7.

e. Instruct subjects to continue eating regular diet

**Baseline visit.**

a. Urine collections will be delivered to the CTSA lab and net acid excretion (NAE), urea, sodium, potassium, chloride, and osmolality of collected urine will be determined.

b. Three-day diet records will be analyzed using Nutrition Data System for Research software version 2011 developed by the Nutrition Coordinating Center, University of Minnesota, Minneapolis, MN. (15) to calculate potential renal acid load (PRAL) of three day diet records. Participants will be instructed on protocol for following the Kansas PKD diet and handouts for diet given. Based on the PRAL determined from dietary records, participants will be educated on what changes need to be made to their baseline diet to be consistent with the Kansas PKD diet guidelines. The Kansas PKD diet will focus on reducing NAE by 50% or more. For example, if a participant has an NAE of ~70mEq/day, they will be instructed on how to reduce net acid excretion to 35mEq/day or less throughincreasing alkaline foods in the diet (fruits and vegetables).

c. Blood pressure, weight, and height will be measured.

d. Blood (10cc) will be drawn and the clot spun to obtain serum for the measurement of baseline creatinine, BUN, sodium, potassium, chloride and bicarbonate levels.

e. A fresh urine sample will be collected (10 cc) in the clinic and tested for biomarkers of ADPKD.

f. Individuals will eat the prescribed KansasPKD diet for the next 4 weeks.

**Phone Call Follow-up**

On day 14 a phone call follow-up will be made with participants to check for compliance to the diet. Participants will report back the amount of fluid, fruits and vegetables (in points), and protein servings they have been eating daily. The researcher will discuss any changes that need to be made to be compliant with the Kansas PKD diet.

**Interim visit.**

a. Participants will collect urine on days 20 and 21of week two following the Baseline visit and take these to the CTSA lab. Net acid excretion (NAE), urea, sodium, potassium, chloride, and osmolality of collected urine will be determined. These samples will be analyzed for NAE and electrolytes the same as at the conclusion of the enrollment period.

b. Diet records will be examined using NDSR to calculate PRAL of three-day diet records (days19, 20, and 21) and estimated NAE determined.

c. Blood pressure, weight, and height will be measured.

d. Blood (10cc) will be drawn and the clot spun to obtain serum for the measurement of baseline creatinine, BUN, sodium, potassium, chloride and bicarbonate levels.

e. Fresh urine sample will be collected (10 cc) in the clinic and tested for biomarkers of ADPKD progression.

**Phone Call Follow-up #2**

On day 28 a phone call follow-up will be made with participants to check for compliance to the diet. Participants will report back the amount of fluid, fruits and vegetables (in points), and protein servings they have been eating daily. The researcher will discuss any changes that need to be made to be compliant with the Kansas PKD diet.

**Final Visit.**

a. Urine collections (days 34 and 35) will be delivered to CTSA lab and NAE, urea, sodium, potassium, chloride, and osmolality of collected urine will be determined.

b. Diet records will be examined using NDSR to calculate PRAL of three-day diet records (days33, 34, and 35) and estimated NAE determined.

c. Blood pressure, weight, and height will be recorded.

d. Blood (10cc) will be drawn and the clot spun to obtain serum for the measurement of baseline creatinine, BUN, sodium, potassium, chloride and bicarbonate levels.

e. Fresh urine sample will be collected (10 cc) in the clinic and tested for biomarkers of ADPKD progression.

f. Participants will complete the Nutritional Hassles Questionnaire at this visit.

g. Semi-structured interviews will also be conducted following the dietary intervention to determine the degree to which individuals found the diet acceptable and the likelihood that they would continue the dietary pattern long term. Interviews will be conducted at the final visit following a semi-structured format, which will allow flexibility to ask open-ended questions and pursue follow-up questions to gain the participants’ perception. All interviews will be audio-taped and transcribed verbatim.

**Analysis of outcomes**

This is a pilot study in which patients serve as their own controls to determine if the Kansas PKD diet lowers amount of net acid excreted than while eating a regular diet.

We will determine descriptive statistics for measurements of serum parameters and urine NAE, electrolytes, creatinine, urea and protein for the baseline, interim and final visits to establish variances. We will determine if baseline urine values determined while eating the regular diet differs from those in interim and final visits by ANOVA and t-test.

a. Mean PRAL will be determined from 3-day diet records of each subject for the Baseline, and Final visits to determine if the dietary acid content was changed by the diet prescription.

b. Mean NAE will be determined from the 2 day urine collections at Baseline, Interim Period and Final visits to determine if the NAE was changed by the diet.

c. I will determine the extent to which changes in diet, PRAL, and urine NAE associate.

d. I will determine the sum of anions and sum of cations in urine and determine if the extent to which the difference between them associates with NAE and PRAL changes.

e. I will determine if participants have reached goal if NAE is decreased 50% from baseline or more.

f. I will determine the degree of diet acceptance based on the analysis of the Nutritional Hassles Questionnaire and the semi-structured interviews.

We acknowledge that we will have some limitations in our statistical analyses. In this pilot study we hope to separate the intra- from the within-individual variability.

**Methods**

**The Kansas PKD Diet.** In accordance with the recent studies implicating excess dietary protein and salt as risk factors for ADPKD progression, the Kansas PKD diet will be limited to 0.8 to 1.0 grams of protein per kilogram body weight and the sodium content to 1.0 to 1.5 mEq/Kg bodyweight. The variable in the diet is the pral and that will be adjusted to reduce the NAE according to the protocol. The remaining minerals, fats, and carbohydrates will conform to current dietary recommendations (16,17). The Kansas PKD diet does not distort the intake of essential nutrients so I will not supplement these participants with vitamins, minerals, or trace substances. Acceptance of the Kansas PKD diet will be determined using Nutrition Hassles Questionnaire and semi-structured interviews. Adherence to Kansas PKD diet will be determined by examining diet records for decreases PRAL and confirming with decreases in urinary acid excretion.

**Urine chemistries.** Urine volume will be measured in the usual manner. Samples will be centrifuged, aliquoted and stored at -80 degrees Celsius in freezers in the Kidney Institute. Net acid excretion will be determined at the Kidney Institute via the Wesson group’s methods (6). Urine electrolytes, urea, creatinine, and osmolality will be measured in the CTSA lab. Urine titratable acidity and MCP1 will be measured in the Kidney Institute using standard methods. All samples will be de-identified. Samples sent to the clinical lab for analysis will not be stored. Samples for the research lab will be store at -80 degrees for up to 5 years in a freezer in the Kidney Institute.

We will collaborate with Jan Simoni, PhD from Texas Tech University with some of the laboratory testing. De-identified samples of plasma and urine will be sent to his lab for analysis of the biomarkers of interest. Dr. Simoni will analyze urine for TGFβ, aldosterone, NAG, NGAL and endothelin. Depending on urine findings, plasma may be analyzed for aldosterone and endothelin.

**Potential difficulties**

This is a pilot study to examine whether the Kansas PKD diet decreases net acid excretion by 50% or more and will be acceptable and sustainable long-term by participants. To our knowledge, this is the first time a diet has been specifically developed to reduce net acid excretion for individuals with ADPKD. These subjects will have life-sustaining renal function and the load of cationic electrolytes derived from fruits and vegetables, specifically potassium, should not be a problem for the kidneys to handle. Since this is the first time a diet of this type has been tested, there may be potential unforeseen variants that were not factored into our Kansas PKD diet protocol. This will be reevaluated at the end of the study to improve our Kansas PKD diet protocol for our future studies. Also, since participants are not being studied in the CTSA with strict dietary and water intake control, failure to adhere to the routine could undermine the expected outcomes.

Adherence is a concern in any study when participants must follow dietary guidelines. Adherence in our study should be demonstrated both by examining diet records and observing a decrease in PRAL and confirmed with decreases in urinary acid excretion. Adherence will be objectively monitored by determining the amounts of urea, sodium, potassium and chloride excreted in the urine in relation to creatinine. We have defined “effective therapy” in the 4 week phase in which the experimental diet is eaten as a decrease in NAE by 50% or more on the 22 day and 36 day 24 h urine collections in comparison to the baseline urine values. There is no published data that forecasts the degree of compliance with a diet of reduced net acid precursors.

**Data and Safety Monitoring:**

There are no data that would suggest that following a diet of reduced net acid precursors would have a negative effect on renal function or be harmful in any other way. Risks from venipuncture and questionnaires are low. The venipunctures will be done by trained professionals, and participants will be able to skip any questions in the questionnaires that might make them uncomfortable. Adverse events are not anticipated, and safety monitoring will be done by the study team. The study team will serve as the data safety and monitoring board.

To protect participants data and uphold confidentiality, recruitment and consenting process will occur in a closed door setting in the clinic. All study procedures will take place in a private patient room and all study records will be stored in a locked location. Study records will be secured in a locked location. Only study personnel will have secured access to these files. Data maintained in a computer database will be password protected. All data collected will be kept strictly confidential and subject will be identified by a unique subject ID number. The code for the ID numbers will be kept separate from the other data and not available to those analyzing the data.

**Literature Cited**

1. Grantham JJ. Autosomal dominant polycystic kidney disease. *New Eng J Med.* 2008;359:1477-1485.
2. Duley I, Gabow P. PKD Patient’s Manual: Understanding and Living with Autosomal Dominant Polycystic Kidney Disease. PKD Foundation. 2006. Available at http://www.pkdcure.org/document.doc?id=99. Accessed on October 7, 2012.
3. Chapman AB, Bost JE, Torres VE, Guay-Woodford L, Bae KT, Landsittel D, Li J, King BF, Martin D, Wetzel LH, Lockhart ME, Harris PC, Moxey-Mims M, Flessner M, Bennett WM, Grantham JJ. Kidney volume and functional outcomes in autosomal dominant polycystic kidney disease. *Clin J Am Soc Nephrol*. 2012;7;479-86.
4. Cowley Jr. BD, Grantham JJ, Muessel MJ, Kraybill AL, Gattone II VH. Modification of Disease Progression in Rats With Inherited Polycystic Kidney Disease. *American Journal of Kidney Diseases.* 1996;27:865-879.
5. Nagao S, Nishii K, Katsuyama M, Kurahashi H, Marunouchi T, Takahashi H, Wallace DP. Increased Water Intake Decreases Progression of Polycystic Kidney Disease in the PCK Rat. *J Am Soc Nephrol.* 2006;17:2220–2227.
6. Goraya N, Simoni J, Jo C, Wesson DE. Dietary acid reduction with fruits and vegetables or bicarbonate attenuates kidney injury in patients with a moderately reduced glomerular filtration rate due to hypertensive nephropathy. *Kidney International.* 2012;81:86-93.
7. Phisitkul S, Khanna A, Simoni J, Broglio K, Sheather S, Rajab MH, Wesson DE. Amelioration of metabolic acidosis in patients with low GFR reduced kidney endothelin production and kidney injury, and better preserve GFR. *Kidney International.* 2010;77:617-623.
8. Mahajan A, Simoni J, Sheather SJ, Broglio KR, Rajab MH, Wesson DE. Daily oral sodium bicarbonate preserves glomerular filtration rate by slowing its decline in early hypertensive nephropathy. *Kidney International.* 2010;78:303-309.
9. Torres VE, Grantham JJ, Chapman AB, Mrug M, Bae KT, King Jr. BF, Wetzel LH, Martin D, Lockhart ME, Bennett WM, Moxey-Mims M, Abebe KZ, Lin Y, Bost, JE. Potentially Modifiable Factors Affecting the Progression of Autosomal Dominant Polycystic Kidney Disease. *Clin J Am Soc Nephrol.* 2011;6:640–647.
10. Remer T. Influence of nutrition on acid-base balance- metabolic aspects. *Eur J Nutr*. 2001;40:214-220.
11. Remer T, Manz F. Estimation of the renal net acid excretion by adults consuming diets containing variable amounts of protein. *Am J Clin Nutr*. 1994; 59:1356-61.
12. Cowley BD, Grantham JJ, Muessel MJ, Kraybill AL, Gattone VH 2^nd^. Modification of disease progression in rats with inherited polycystic kidney disease. *Am J Kidney Dis.* 1996;27(6):865-879.
13. Tanner GA, Tanner JA. Citrate therapy for polycystic kidney disease in rats. *Kidney International.* 2000;58:1859-1869.
14. Grantham JJ. Autosomal Dominant Polycystic Kidney Disease. *NEJM.* 2008;359:1477-1485.
15. Schakel SF. Maintaining a nutrient database in a changing marketplace: Keeping pace with changing food products - A research perspective. J Food Comp and Anal. 2001;14:315-322.
16. Dietary Reference Intakes for Energy, Carbohydrate. Fiber, Fat, Fatty Acids, Cholesterol, Protein, and Amino Acids (2002/2005). Food and Nutrition Board, Institute of Medicine, National Academies. Available at http://www.iom.edu/Global/News%20Announcements/~/media/C5CD2DD7840544979A549EC47E56A02B.ashx. Accessed on October 8, 2012.
17. Dietary Reference Intakes (DRIs): Recommended Dietary Allowances and Adequate Intakes, Vitamins. Food and Nutrition Board, Institute of Medicine, National Academies, 2012. Available at http://www.iom.edu/Global/News%20Announcements/~/media/C5CD2DD7840544979A549EC47E56A02B.ashx. Accessed on October 8, 2012.
