# Supplementary material for: Experiences and Perspectives of Polycystic Kidney Disease Patients following a Diet of Reduced Osmoles, Protein, and Acid Precursors Supplemented with Water: A Qualitative Study
Source: PLoS One. 2016 Aug 18;11(8):e0161043. doi: 10.1371/journal.pone.0161043 (PMC4990231; doi:10.1371/journal.pone.0161043)
Supplement: S1 Table — Abbreviations: PKD, polycystic kidney disease. (DOCX) [file pone.0161043.s004.docx]

| S1 Table. Semi-structured interview questions | | | |
| --- | --- | --- | --- |
| **Topics** | **Main Question** | **Follow-up Question** | **Probes** |
| Kansas PKD Diet | Tell me about your overall experience with the Kansas PKD Diet | What did you feel like were the biggest changes from your usual diet that you had to make to follow the Kansas PKD diet?  What aspects of the diet were the most enjoyable?  What components of the diet were easiest to follow?  What components of the diet were most difficult to follow?  Tell me about times when you were not able to follow the Kansas PKD Diet (see probing question) | When you were not able to follow the diet, what were the barriers? |
| Point System (Fruits/Vegetables) | Tell me how easy/difficult it was to use the fruits/vegetables point system handouts to track your points (see probing question) | How well did you understand the fruits/vegetables point system?  What aspects of the point system would you change?  What aspects of the point system would you absolutely NOT change?  Did you feel like getting the required amount of points each day was a challenge? And was it a realistic goal? | What made it difficult to follow? |
| Point System (Protein) | Tell me how easy/difficult it was to use the protein points system (see probing question) | How well did you understand the protein point system?  What aspects of the point system would you change?  What aspects of the point system would you absolutely NOT change?  Did you feel like staying below the point restriction each day was a challenge? And was it a realistic goal? | What made it difficult to follow? |
| Sodium | Tell me how easy/difficult it was to reduced sodium in the diet | How did you go about reducing your sodium intake?  Did you feel like doing these things each day was a challenge? And was it a realistic goal? |  |
| Fluids | Tell me how easy/difficult it was to meet your fluid needs | How did you go about tracking your fluid intake?  Did you feel like meeting your fluid goal was a challenge? And was it a realistic goal? |  |
| Lifestyle | How did following this diet affect other aspects of your life? | Describe your experience grocery shopping while on this diet?  Describe your experiences eating out while on this diet?  Were there any difficulties financially while following this diet (assume there was no stipend)?  Did you feel you had the necessary food preparation skills and knowledge to follow this diet?  Did you feel following the Kansas PKD diet required any additional time commitments?  Did following the Kansas PKD diet affect any other obligations in your life? |  |
| Future | Now that you have completed the study, how would you use this information in the future? | When you think about the Kansas PKD diet, what is the probability you would follow this diet in the future?  How would you feel about continuing to track your Fruits/vegetable points and eating the prescribed points?  How would you feel about continuing to track your protein points and continuing to restrict your points?  How would you feel about continuing to restrict your sodium intake?  How would you feel about continuing to drink the prescribed amount of water?  How would you feel about following this diet over the course of your life?  What might prevent you from eating in this manner?  What help/support would be needed to help you follow:  -Fluid requirements?  -Reducing sodium intake?  -Eating more fruits and vegetables?  -Eating less protein (meat, dairy, etc)? | If you are not likely to continue following this diet, are their certain components of the diet you may continue to follow? |
| Suggestions | What do you feel we can improve? | What aspects of the diet would you change to make it easier to follow?  What aspects of the diet would you keep? |  |

Abbreviations: PKD, polycystic kidney disease.
